# Effects of instability direction on early neuromuscular responses of trunk and hip muscles during rapid squatting

**DOI:** 10.3389/fphys.2026.1873134

**Published:** 2026-09-01

**Authors:** Kohei Yoshikawa, Matsuri Hashimoto, Shinichi Noguchi, Noriyuki Kida

**Affiliations:** 1 Department of Rehabilitation, Kanazawa Orthopaedic and Sports Medicine Clinic, S, Ritto, Shiga, Japan; 2 Graduate School of Science and Technology, Kyoto Institute of Technology, Kyoto, Japan; 3 Department of Physical Therapy, Biwako Professional University of Rehabilitation, Higashiomi, Shiga, Japan; 4 Faculty of Arts and Sciences, Kyoto Institute of Technology, Kyoto, Japan

**Keywords:** core stability, directional uncertainty, dynamic postural stability, neuromuscular response, postural control

## Abstract

**Introduction:**

Evidence suggests that unstable support-surface conditions alter trunk muscle activity during exercise. However, previous studies predominantly focused on the overall magnitude of muscle activity. Consequently, the effect of instability direction on the temporal characteristics of early neuromuscular responses during dynamic tasks remains poorly understood. This study investigated the effects of anteroposterior (AP) and mediolateral (ML) instabilities on early neuromuscular responses in the trunk and hip muscles during rapid squatting.

**Methods:**

Twenty healthy adult men performed rapid squats in a within-subject repeated-measures design under AP, ML, and stable floor (ST) conditions. The AP and ML conditions were created by orienting a uniaxial rocker board to permit rocking in the respective directions. The auditory cue was presented at a random time between 4.0 and 6.0 s after trial initiation. Surface electromyography (EMG) was used to record muscle activity in the internal oblique/transversus abdominis (IO/TrA), external oblique (EO), rectus abdominis (RA), erector spinae (ES), gluteus medius (GMed), and gluteus maximus (GMax). Primary outcomes were EMG onset latency and early muscle activity (%MVC) during the first 0–75 ms after EMG onset, representing the initial phase of neuromuscular activation with relatively limited influence of sensory feedback. Data were analyzed using two-way repeated-measures analysis of variance (ANOVA) (condition × muscle).

**Results:**

For EMG onset latency, significant main effects of condition and muscle were observed; however, no significant interaction was observed. *Post hoc* comparisons indicated that the onset latency was significantly shorter in the AP condition than in the ST condition. Early muscle activity showed significant main effects of condition and muscle and a significant condition × muscle interaction. RA activity was significantly lower in the AP and ML conditions than in the ST condition. No significant differences were found in the other muscles.

**Discussion:**

These findings provide limited evidence for direction-specific responses because no clear AP–ML differences were observed. Muscle-specific differences in early activity were mainly limited to lower RA activity under both unstable conditions relative to the ST condition.

## 1 Introduction

Humans must perform movements while maintaining postural stability under the perturbations and unstable support-surface conditions encountered during daily activities and sports (Horak, 2006; Massion, 1992; Peterka, 2002). Postural control is a complex sensorimotor process aimed at achieving postural orientation and equilibrium, and is governed by the interaction between biomechanical constraints and neural control mechanisms (Horak, 2006). Within this framework, it is well established that feedforward processes (anticipatory postural adjustments) and feedback processes (reactive or reflex mechanisms) operate complementarily to counteract self-induced or external perturbations (Aruin and Latash, 1995; Horak, 2006; Massion, 1992; Peterka, 2002). Consequently, the success of postural control likely depends on the temporal organization of neuromuscular responses within the critical time window surrounding movement initiation. In the present study, instability refers specifically to mechanical instability of the support surface created by a uniaxial rocker board. The anteroposterior (AP) and mediolateral (ML) conditions represent the directions in which the board was permitted to rock, rather than externally applied perturbations or the direction of body sway.

Most studies on unstable support surfaces have focused on amplitude-based measures, such as mean or integrated electromyographic (EMG) activity (Andersen et al., 2014; Anderson and Behm, 2005; Behm et al., 2002; Bressel et al., 2009; Calatayud et al., 2015; Moosaei Saein et al., 2024; Prieske et al., 2013; Saeterbakken and Fimland, 2013; Wahl and Behm, 2008; Youdas et al., 2015). Studies specifically examining squatting have shown that unstable support-surface conditions can influence muscle activity and force output, although the magnitude and direction of these effects vary according to the instability device, loading condition, and examined muscle (Andersen et al., 2014; Anderson and Behm, 2005; Saeterbakken and Fimland, 2013). However, these studies generally evaluated average or integrated EMG activity rather than EMG onset timing or early muscle activity immediately after onset. To our knowledge, no previous study has examined EMG onset latency and early muscle activity during rapid squatting while directly comparing AP and ML rocking on a uniaxial rocker board.

Previous studies on stable surfaces have demonstrated that trunk EMG onset timing and coordination patterns are task dependent (Granata and England, 2006; Hodges and Richardson, 1996; Moseley et al., 2002; Saito et al., 2023a, 2023; Saunders et al., 2004; Silfies et al., 2009). Studies using external postural perturbations have also shown that strategy selection, such as ankle-versus-hip strategies, depends on the nature and direction of the destabilizing force (Duchene et al., 2021; Horak and Nashner, 1986; Vera-Garcia et al., 2006). AP stability during quiet standing is regulated primarily by the ankle strategy, whereas ML stability relies more heavily on the hip strategy (Winter et al., 1996). Furthermore, muscle synergies in response to perturbations have been reported to differ between the AP and ML directions (Ting and McKay, 2007). Although these findings provide a basis for considering direction-specific postural control, externally applied perturbations differ from the unstable support surface used in the present study. Therefore, it remains unclear whether the direction of rocking permitted by a uniaxial support surface influences early neuromuscular responses during dynamic tasks.

Our previous work using lower-limb tasks in the supine position, including the Active Straight Leg Raise (ASLR) and the Sahrmann Core Stability Test (SCST), indicated that movement speed might modulate trunk muscle activation patterns (Yoshikawa et al., 2025, 2024). These findings motivated us to examine movement speed as a temporal constraint and to focus on the temporal characteristics of muscle recruitment. To extend this question to a standing whole-body task, we selected rapid bilateral squatting as a controlled dynamic task involving coordinated trunk and hip activity. The rapid movement requirement was used to emphasize the temporal organization of neuromuscular responses around movement initiation, whereas the bilateral squat allowed the movement target and base of support to be standardized across support-surface conditions. Thus, this task was considered suitable for examining EMG onset latency and early muscle activity under AP and ML support-surface instability.

To characterize responses across muscles with different functional roles during rapid squatting, we selected trunk and hip muscles involved in postural control. The rectus abdominis (RA) and erector spinae (ES) were included as muscles related to sagittal-plane trunk control, whereas the external oblique (EO) and gluteus medius (GMed) were included because of their contributions to lateral trunk and frontal-plane hip control, respectively. The internal oblique/transversus abdominis (IO/TrA) was included because of its generalized role in trunk stabilization, and the gluteus maximus (GMax) because of its contribution to hip extension during squatting. Accordingly, AP rocking was expected to place greater demands on sagittal-plane trunk control, whereas ML rocking was expected to place greater demands on lateral trunk and frontal-plane hip control.

The primary objective of this study was to determine whether support-surface condition (AP instability, ML instability, and stable condition) influenced EMG onset latency and early muscle activity during the first 0–75 ms after EMG onset in the trunk and hip muscles during rapid squatting. We hypothesized that these responses would vary according to the support condition and muscle. Our secondary *a priori* hypotheses concerned muscle-specific directional responses. Specifically, we hypothesized that early muscle activity would be greater in the RA and ES under AP instability and in the EO and GMed under ML instability. We further hypothesized that the IO/TrA would show relatively early activation regardless of the direction of instability, reflecting its generalized role in trunk stabilization.

## 2 Methods

### 2.1 Participants

Twenty healthy adult men participated in this study. The inclusion criteria were male sex and age 20 years or older. The exclusion criteria were as follows: (1) history of surgery, (2) limited joint range of motion, (3) marked balance impairment, (4) severe cardiac disease, and (5) skin abnormalities at the electrode placement sites. The sample size was calculated *a priori* using G*Power software (version 3.1). The primary analysis was designed to detect a condition (three levels) × muscle (six levels) interaction. Assuming an effect size of f = 0.30, statistical power of 0.80, significance level of 0.05, three conditions, and correlation of 0.5 among repeated measures, the required sample size was estimated to be 20 participants.

This study was approved by the Ethics Committee for Human Sciences Research at the Kyoto Institute of Technology (approval No. 2025-103). All participants received an explanation of the study purpose and procedures and provided written informed consent prior to participation.

### 2.2 Experimental design

A within-subjects, repeated-measures design was used. The experimental conditions consisted of AP, ML, and stable floor (ST) conditions, with unstable conditions created using a uniaxial rocker board. Each participant performed seven trials under each condition, for a total of 21 trials. The order of the conditions and trials was randomized across participants. Rest intervals of 30–60 s were provided between trials and 2–3 min were provided between conditions.

### 2.3 Experimental apparatus

**Figure 1:**
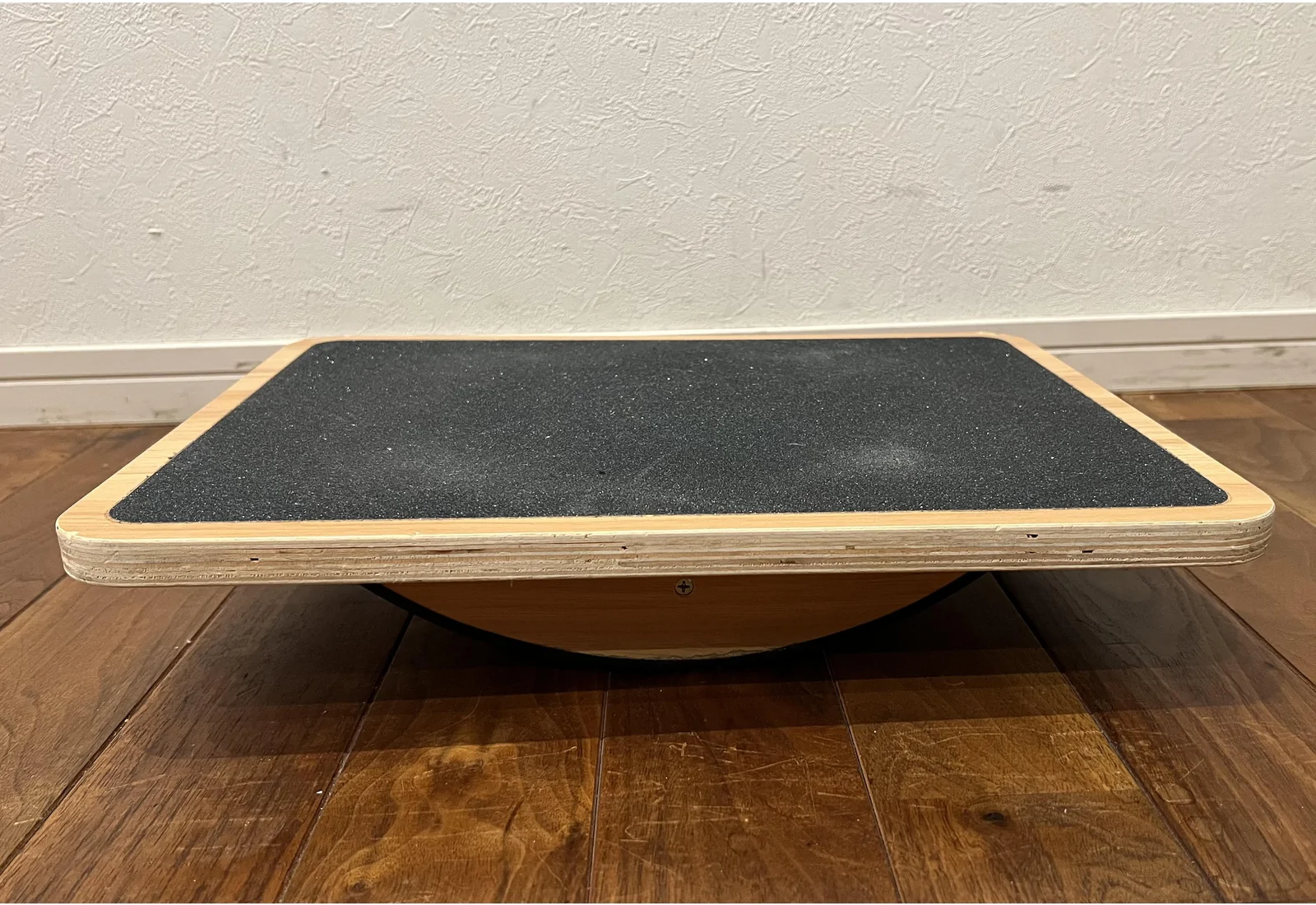
Uniaxial rocker board used to create unstable standing conditions.

A uniaxial wooden rocker board measuring 43 cm in length, 33 cm in width, and 8 cm in height (Professional Wooden Balance Board, STRONGTEK, Sugar Land, TX, USA) was used to create the unstable support-surface conditions (**Figure 1**). The board consisted of a rectangular standing platform mounted on a semicircular base and permitted a maximum tilt of approximately 15° to either side about a single rocking axis. For the AP condition, the board was oriented to permit rocking in the participant’s anteroposterior direction; for the ML condition, it was rotated by 90° to permit rocking in the mediolateral direction. The same board was used in both conditions so that the permitted rocking axis could be manipulated while maintaining the other board characteristics. Thus, AP and ML denote the mechanically permitted directions of board rocking rather than measured directions of body sway. In the ST condition, the task was performed while the participant stood on the floor.

### 2.4 Task

The task consisted of performing a rapid squat in response to an auditory cue. The participants first maintained a standing posture for 3 seconds with the foot width set at 90% of the distance between the anterior superior iliac spines (ASIS). They were then instructed to squat as quickly as possible to a target knee flexion angle of 70° ± 5° in response to the cue and to maintain that position for 1–2 s. Movement execution speed was not standardized using a metronome or target duration, allowing each participant to perform the cue-triggered squat at their maximal voluntary speed. The IMU was used to identify movement onset but not to quantify movement duration or angular velocity. The auditory cue was presented at a random time between 4.0 and 6.0 s in each trial.

**Figure 2:**
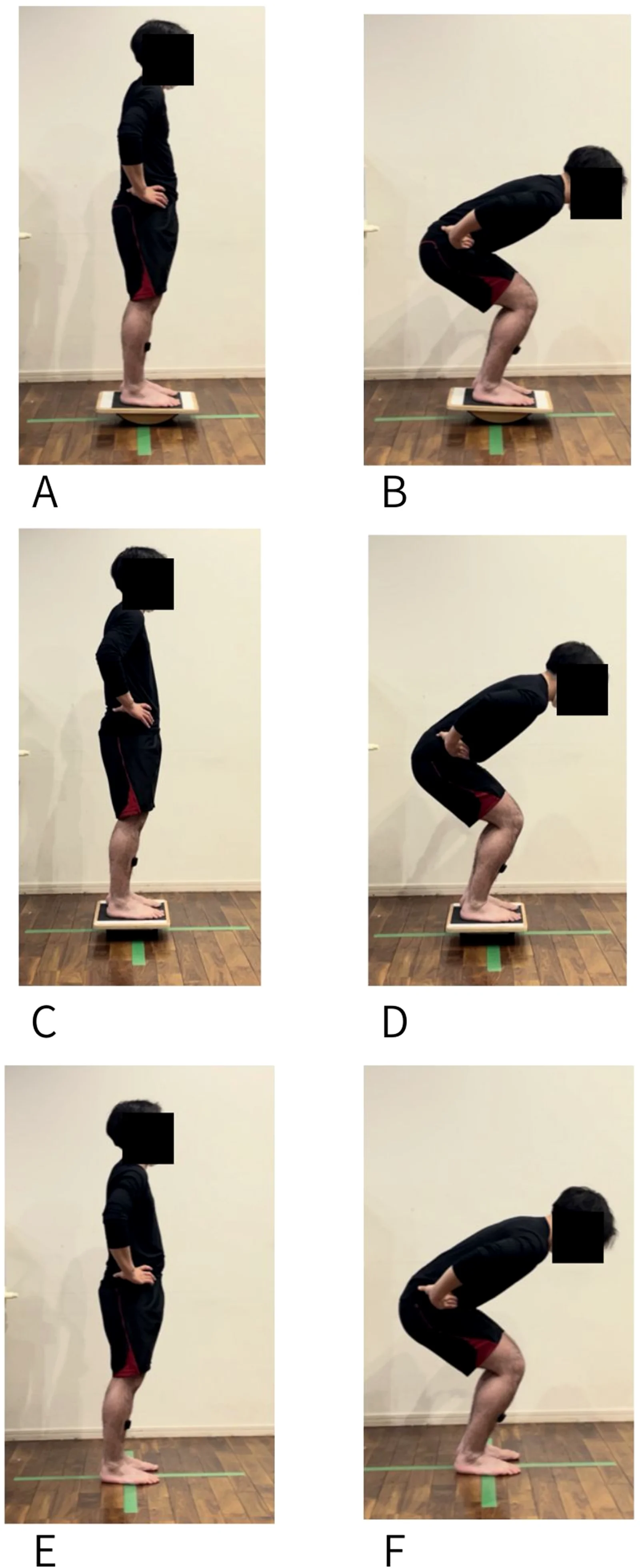
Experimental conditions and squat task performance. The starting and end positions of the rapid squat task are shown for each condition: anteroposterior instability **(A, B)**, medial–lateral instability **(C, D)**, and stable floor condition **(E, F)**. The participants placed their hands on the iliac crests and the target knee flexion angle was set at 70° to standardize task performance.

To standardize movement performance, the participants placed both hands on the iliac crests to prevent arm swing, heel lift-off was not permitted, and forward knee displacement was restricted to within 50% of the foot length. Reproducibility of the target knee angle was sufficiently established during practice before the experimental trials (**Figure 2**).

### 2.5 Measurements

#### 2.5.1 Surface electromyography

Surface EMG was used to record the activity of the trunk and hip muscles on the dominant side. The dominant side was defined as the preferred leg used to kick a ball. Based on this criterion, the right side was dominant in all 20 participants. A Delsys system (Trigno Avanti System, Delsys Inc., Natick, MA, USA) was used for data acquisition. Six muscles were examined: the internal oblique/transversus abdominis (IO/TrA), external oblique (EO), rectus abdominis (RA), erector spinae (ES), gluteus medius (GMed), and gluteus maximus (GMax). Before electrode placement, the skin was shaved, abraded, cleaned with alcohol, and dried to reduce skin impedance to below 5 kΩ. Wireless sensors were placed parallel to the muscle fiber direction at the following locations: IO/TrA, 1 cm medial and 0.5 cm inferior to the horizontal line from the ASIS (Marshall and Murphy, 2003); EO, 1 cm superior to the horizontal line through the umbilicus and 1 cm lateral to the lateral border of the RA muscle belly (McGill and Norman, 1986); RA, 1 cm superior to the umbilicus and 2 cm lateral to the midline (Beales et al., 2009; McGill and Norman, 1986); ES, 3 cm lateral to the L3 spinous process (Hermens et al., 2000); GMed, at 50% of the distance from the iliac crest to the greater trochanter (Hermens et al., 2000); and GMax, at the midpoint between the second sacral vertebra (S2) and the greater trochanter (Hermens et al., 2000). For IO/TrA electrode placement, ultrasonography was used to confirm muscle location and fiber orientation to avoid overlapping with the EO and improve recording accuracy (Marshall and Murphy, 2003; Silfies et al., 2009). Care was also taken to optimize electrode placement and alignment with muscle fibers in all target muscles to minimize crosstalk with adjacent muscles (McGill and Karpowicz, 2009). The EMG signals were sampled at 2,148 Hz.

#### 2.5.2 Identification of movement onset using an inertial sensor

Movement onset (T0) was identified using angular velocity signals from an inertial measurement unit (IMU) attached to the sacral region. The IMU signals were sampled at a frequency of 370 Hz. The vector magnitude of the three-axis angular velocity signal (|Gyro|) was calculated and smoothed using a fourth-order low-pass Butterworth filter at 5 Hz. The baseline period was defined as 3.0–4.0 s. The threshold was set at the mean + 4 standard deviations (SD) of this baseline period. The 4-SD threshold was selected based on preliminary pilot testing of different threshold levels. Compared with the 3-SD threshold, the 4-SD criterion reduced false-positive detections caused by baseline fluctuations and noise and most consistently corresponded to visually identifiable movement onset. The same threshold was applied consistently across all participants and trials. T0 was defined as the first time point after 4.0 s at which the smoothed |Gyro| continuously exceeded the threshold for at least 50 ms.

#### 2.5.3 Visual confirmation of trial validity

For each trial, the EMG and IMU waveforms were visually inspected, and the detected movement onset (T0) and EMG onset times for each muscle were recorded. When necessary, the timing of onset was corrected according to the predefined procedures described below.

### 2.6 Data processing

#### 2.6.1 EMG signal processing

The missing EMG data points were linearly interpolated. The EMG signals were then processed using a fourth-order Butterworth bandpass filter (20–500 Hz), followed by full-wave rectification. A smoothed linear envelope was subsequently generated using a zero-phase fourth-order low-pass Butterworth filter at 20 Hz. The 20-Hz cutoff was selected based on preliminary pilot testing. This setting reduced noise-related fluctuations while preserving the rapid increase in EMG activity used for onset detection. The zero-phase implementation was used to avoid phase delay.

#### 2.6.2 Determination of EMG onset

EMG onset for each muscle was determined using the EMG linear envelope relative to T0. The baseline period was defined as 700–400 ms before T0 in each trial. For each muscle, the mean and SD during this baseline period were calculated, and the threshold was set at the mean + 3 SD. EMG onset was defined as the first time point at which the EMG envelope exceeded this threshold continuously for at least 50 ms. EMG onset latency was calculated as the difference between this time point and T0 (onset time - T0), and negative values were allowed. This procedure was based on previously established threshold-based criteria for determining EMG onset (Hodges and Bui, 1996).

#### 2.6.3 Manual correction of EMG onset

The time window from 0 to 75 ms after the onset of the EMG was adopted as the evaluation window for early muscle activity. This interval was selected because the initial phase, within 50–75 ms after the activation onset, is considered to reflect early neuromuscular responses, including motor unit recruitment and discharge rate, with less influence from voluntary feedback corrections and muscle mechanical properties that become more evident after approximately 100 ms (Folland et al., 2014; Maffiuletti et al., 2016). In this study, this time window was intended to capture early neuromuscular responses with relatively limited influence of sensory feedback. This automated threshold-based procedure was consistent with previous threshold-based approaches for EMG onset detection (Hodges and Bui, 1996; Tenan et al., 2017). Automatically detected EMG onset times were visually inspected in all trials by a single assessor. Manual correction was performed only when the automated threshold crossing was judged to reflect transient noise, baseline fluctuation, or an isolated spike rather than a sustained increase in EMG activity. In such cases, onset was corrected to the first time point at which a sustained increase in EMG activity was observed. The same criteria were applied consistently across all participants, conditions, and muscles. Manual correction was required in 14.3% of trials. In addition, a randomly selected subset of trials was re-evaluated by the same assessor as a procedural check of intra-rater consistency.

#### 2.6.4 Calculation of early muscle activity after EMG onset (%MVC)

The muscle activity was normalized to the maximum voluntary contraction (MVC) value for each muscle. The MVC was measured using standardized manual resistance tasks based on previously published methods for abdominal and trunk muscle EMG normalization (Beith et al., 2001; Vera-Garcia et al., 2010). Each MVC task was performed for 5 s and repeated twice with sufficient rest between trials. The highest EMG value obtained during the MVC trials was used for normalization. Using the final accepted EMG onset time, muscle activity during the 0–75 ms interval after EMG onset was calculated for each muscle, divided by the MVC value, and expressed as a percentage (%MVC). This served as an index of early muscle activity for each muscle. Because early muscle activity values showed a right-skewed distribution, log10 transformation was applied to improve normality before statistical analysis. Normality was assessed using the Shapiro–Wilk test. Because the EMG envelope was rectified and smoothed, all early muscle activity values were positive, and no zero values were observed; therefore, no constant was added before log transformation. Statistical analyses for early muscle activity were performed using log10-transformed %MVC values, whereas descriptive statistics and figures are presented in the original %MVC scale to facilitate interpretation.

### 2.7 Outcome measures

The primary outcome measures were EMG onset latency for each muscle (relative to T0, in ms) and early muscle activity during the 0–75 ms interval after the final accepted EMG onset, expressed as %MVC.

### 2.8 Statistical analysis

For each condition, the mean value of the valid trials was used for the analysis. EMG onset latency and early muscle activity during the 0–75 ms period after EMG onset (%MVC) were used as dependent variables. Two-way repeated-measures analysis of variance (ANOVA) was performed with the condition (AP, ML, and ST) and muscle (six muscles) as within-subject factors. For early muscle activity (%MVC), log10-transformed values were used for statistical analysis to improve normality. Statistical analyses of early muscle activity, including estimated mean differences and 95% confidence intervals, were based on log10-transformed data, whereas descriptive statistics and figures are presented in the original %MVC scale. The main effects of the condition and muscle as well as the condition × muscle interactions were examined. Sphericity was assessed using Mauchly’s test, and when the assumption of sphericity was violated, the Greenhouse–Geisser correction was applied.

*Post hoc* comparisons were performed using the Bonferroni method when a significant interaction or main effect was observed. The same statistical models were applied to all examined muscles and support-surface conditions. Muscle-specific interpretations were guided by the *a priori* hypotheses described in the Introduction, but no separate *post hoc* selection of muscles was performed. Estimated differences and 95% confidence intervals were considered for all relevant pairwise comparisons. Statistical significance was set at p < 0.05.

## 3 Results

### 3.1 Participant characteristics

Participant characteristics are presented in **Table 1**. The mean age, height, and body mass of the participants were 27.6 ± 6.5 years, 173.5 ± 7.5 cm, and 67.4 ± 9.0 kg, respectively.

**Table 1:** Participant characteristics.

Variable	n	Mean ± SD
Age (years)	20	27.6 ± 6.5
Height (cm)	20	173.5 ± 7.5
Body mass (kg)	20	67.4 ± 9.0

Values are expressed as the mean ± standard deviation (SD). n, Number of participants.

### 3.2 Representative EMG and IMU waveforms across conditions

**Figure 3:**
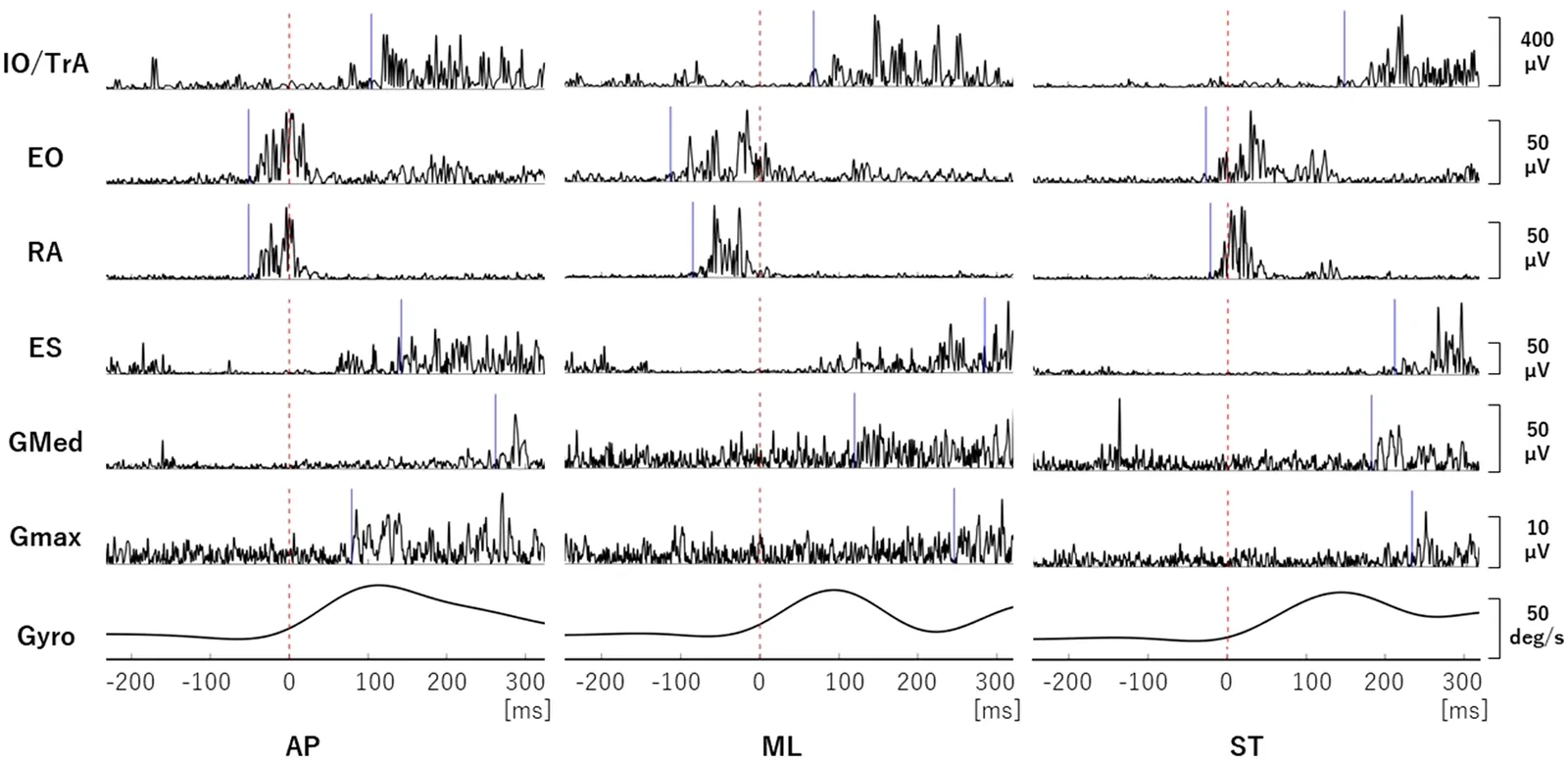
Representative EMG and IMU waveforms for each condition. The raw EMG and smoothed |Gyro| waveforms from a representative participant illustrate the temporal relationship between movement initiation and muscle activation. The vertical red dotted line indicates movement onset (T0), which was determined from the angular velocity signal shown in the bottom row. Vertical blue solid lines indicate the detected EMG onset for each muscle. The interval between the red and blue lines represents EMG onset latency. AP, anteroposterior instability; ML, mediolateral instability; ST, stable floor condition.

The representative EMG and IMU waveforms for each condition are shown in **Figure 3**. These waveforms represent data from a single participant and illustrate the temporal relationship between movement onset (T0), as identified by the IMU, and the onset of muscle activity in each muscle.

### 3.3 EMG onset latency

The results of the EMG onset latency across the conditions are presented in **Table 2**. Mauchly’s test of sphericity indicated that the assumption of sphericity was met for the main effect of condition (p = 0.058). However, sphericity was violated for the main effect of muscle and the condition × muscle interaction; thus, the Greenhouse–Geisser correction was applied. The main effect of condition was significant (F (2, 38) = 3.59, p = 0.037, ηp² = 0.16), as was the main effect of muscle (F (3.07, 58.30) = 52.06, p < 0.001, ηp² = 0.73). The condition × muscle interaction was not significant (F (5.15, 97.87) = 1.25, p = 0.293, ηp² = 0.06) (**Table 3**).

**Table 2:** EMG onset latency for each muscle across conditions [mean ± standard deviation (SD), ms].

Condition	IO/TrA	EO	RA	ES	GMed	GMax
AP	25.7 ± 74.2	-55.1 ± 39.1	-32.1 ± 82.9	102.7 ± 56.6	104.4 ± 50.2	162.9 ± 74.2
ML	36.4 ± 74.9	-48.8 ± 62.0	-46.3 ± 82.5	104.6 ± 64.8	121.9 ± 59.0	189.4 ± 70.5
ST	53.7 ± 64.7	-21.7 ± 97.1	-27.4 ± 89.9	128.8 ± 52.5	118.1 ± 62.2	187.9 ± 67.3

Values represent the time difference relative to the movement onset (T0), and negative values indicate muscle activation preceding T0. IO/TrA, internal oblique/transversus abdominis; EO, external oblique; RA, rectus abdominis; ES, erector spinae; GMed, gluteus medius; GMax, gluteus maximus. AP, anteroposterior instability; ML, mediolateral instability; ST, stable floor condition.

**Table 3:** Results of the two-way repeated-measures ANOVA for EMG onset latency.

Effect	Sphericity correction	df	F	p	Partial η²
Condition	Assumed	2, 38	3.59	0.037*	0.16
Muscle	Greenhouse–Geisser	3.07, 58.30	52.06	<0.001***	0.73
Condition × Muscle	Greenhouse–Geisser	5.15, 97.87	1.25	0.293	0.06

df, degrees of freedom; F, F-statistic; p, p-value; ηp², partial eta squared. Sphericity correction was applied using the Greenhouse–Geisser method when the assumption of sphericity was violated (Mauchly’s test, p < 0.05). “Assumed” indicates that the assumption of sphericity was met. Significant effects are indicated by asterisks: *P < 0.05, ***P < 0.001.

For the main effect of condition, Bonferroni-adjusted comparisons showed that EMG onset latency was significantly shorter in the AP condition than in the ST condition (mean difference = -22 ms, p = 0.040, Cohen’s dz = -0.61). No significant differences were observed between the ML and ST conditions (mean difference = -14 ms, p = 0.560, Cohen’s dz = -0.31) or between the AP and ML conditions (mean difference = -8 ms, p = 0.629, Cohen’s dz = -0.29) (**Table 4A**).

**Table 4:** Bonferroni-adjusted *post hoc* comparisons for EMG onset latency.

A. Pairwise comparisons for the main effect of condition				
Comparison	Mean difference (I - J)	95% Confidence interval	P-value	Cohen’s dz
AP vs ST	-22	-43 to -1	0.040*	-0.61
ML vs ST	-14	-40 to 13	0.560	-0.31
AP vs ML	-8	-25 to 8	0.629	-0.29

B. Condition comparisons for each muscle					
Muscle	Comparison	Mean difference (I - J)	95% Confidence interval	P-value	Cohen’s dz
IO/TrA	AP vs ST	-28	-53 to -3	0.025*	-0.66
ES	AP vs ST	-26	-48 to -4	0.019*	-0.69

Mean differences and 95% confidence intervals are expressed in milliseconds (ms). Cohen’s dz represents the standardized mean difference for paired comparisons. IO/TrA, internal oblique/transversus abdominis; ES, erector spinae. AP, anteroposterior instability; ML, mediolateral instability; ST, stable floor condition. Significant differences are indicated by asterisks; *p < 0.05.

As the condition × muscle interaction was not significant, muscle-specific comparisons were treated as secondary observations. In these comparisons, onset latency was shorter in the AP condition than in the ST condition for the IO/TrA (mean difference = -28 ms, p = 0.025, Cohen’s dz = -0.66) and ES (mean difference = -26 ms, p = 0.019, Cohen’s dz = -0.69) (**Table 4B**). These muscle-specific results should therefore be interpreted cautiously.

### 3.4 Early muscle activity during the first 0–75 ms after EMG onset

**Figure 4:**
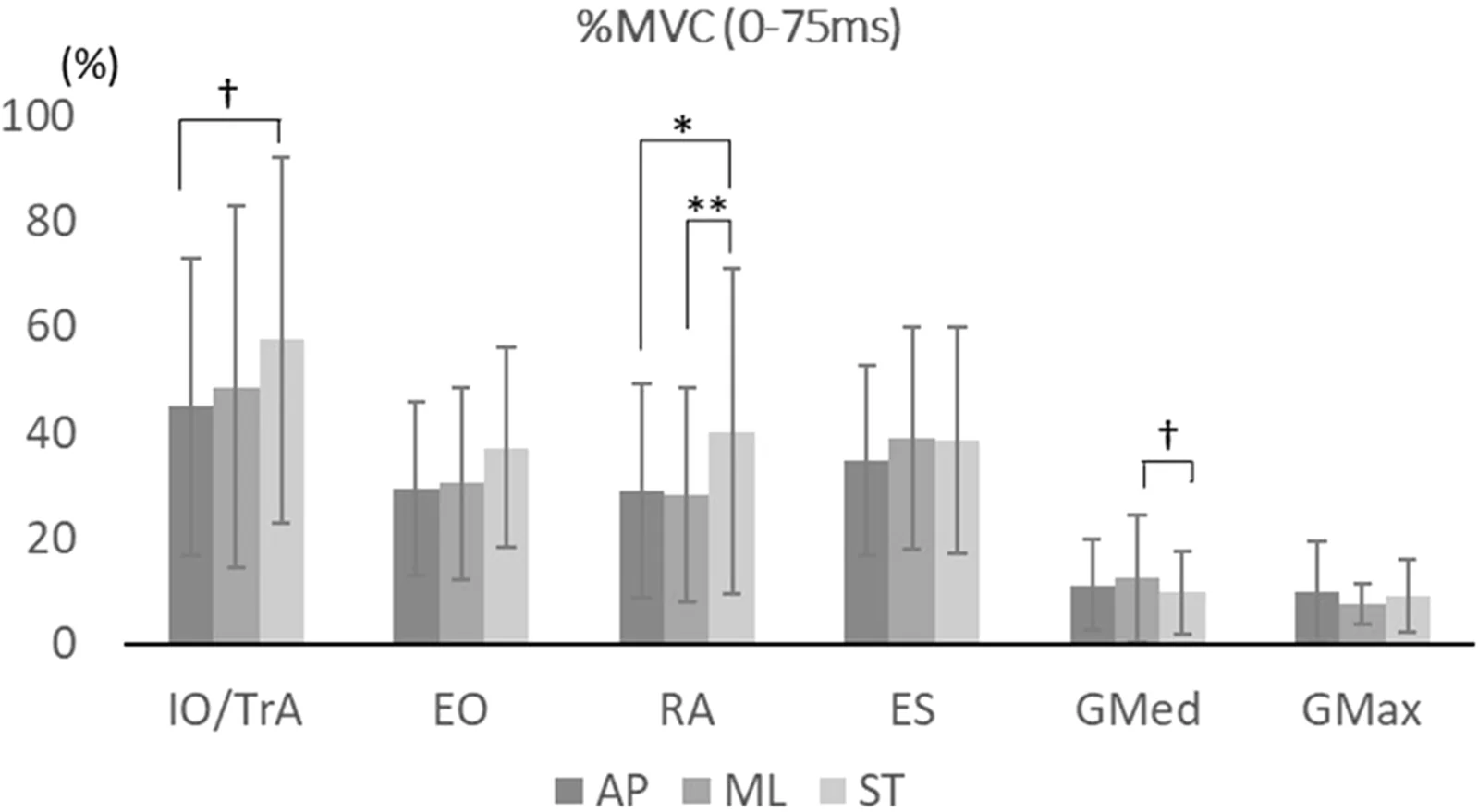
Early muscle activity for each muscle across conditions. The bars represent the mean muscle activity during the first 0–75 ms after EMG onset, expressed as %MVC. Error bars indicate standard deviation. Significant differences between conditions within each muscle were determined using Bonferroni-adjusted *post hoc* comparisons based on log10-transformed data. Significant differences are indicated by asterisks (*p < 0.05, **p < 0.01). Daggers indicate non-significant pairwise comparisons with p values between 0.05 and 0.10. AP, anteroposterior instability; ML, mediolateral instability; ST, stable floor condition.

The results for early muscle activity during the first 0–75 ms after EMG onset (%MVC) are summarized in **Table 5**. Mauchly’s test indicated that the assumption of sphericity was met for the main effects of the condition (p = 0.089) and muscle (p = 0.646). Sphericity was violated for the condition × muscle interaction; therefore, the Greenhouse–Geisser correction was applied. Significant main effects were observed for condition (F (2, 38) = 4.63, p = 0.016, ηp² = 0.20) and muscle (F (5, 95) = 31.00, p < 0.001, ηp² = 0.62). The condition × muscle interaction was also significant (F (5.35, 101.70) = 2.72, p = 0.021, ηp² = 0.13) (**Table 6**). Comparisons of early muscle activity for each muscle across conditions are shown in **Figure 4**.

**Table 5:** Early muscle activity for each muscle across conditions [mean ± standard deviation (SD), %MVC].

Condition	IO/TrA	EO	RA	ES	GMed	GMax
AP	45.0 ± 28.2	29.6 ± 16.4	29.0 ± 20.2	34.8 ± 17.9	11.1 ± 8.6	9.9 ± 9.5
ML	48.7 ± 34.3	30.4 ± 18.3	28.2 ± 20.3	39.0 ± 21.0	12.5 ± 12.0	7.6 ± 3.7
ST	57.6 ± 34.6	37.1 ± 19.0	40.2 ± 30.9	38.6 ± 21.5	9.8 ± 7.9	9.2 ± 7.0

Values represent muscle activity during the first 0–75 ms after EMG onset normalized to maximum voluntary contraction (%MVC). IO/TrA, internal oblique/transversus abdominis; EO, external oblique; RA, rectus abdominis; ES, erector spinae; GMed, gluteus medius; GMax, gluteus maximus. AP, anteroposterior instability; ML, mediolateral instability; ST, stable floor condition.

**Table 6:** Results of the two-way repeated-measures ANOVA for early muscle activity during the first 0–75 ms after EMG onset.

Effect	Sphericity correction	df	F	p	Partial η²
Condition	Assumed	2, 38	4.63	0.016*	0.20
Muscle	Greenhouse–Geisser	5, 95	31.00	<0.001***	0.62
Condition × Muscle	Greenhouse–Geisser	5.35, 101.70	2.72	0.021*	0.13

df, degrees of freedom; F, F-statistic; p, p-value; ηp², partial eta squared. Sphericity correction was applied using the Greenhouse–Geisser method when the assumption of sphericity was violated (Mauchly’s test, p < 0.05). “Assumed” indicates that the assumption of sphericity was met. Significant effects are indicated by asterisks: *P < 0.05, ***P < 0.001. Log10-transformed values were used for the statistical analyses to improve normality.

For the main effect of condition, Bonferroni-adjusted comparisons showed no significant differences between the AP and ST conditions (mean difference = -0.059, p = 0.074, Cohen’s dz = -0.55), between the ML and ST conditions (mean difference = -0.050, p = 0.110, Cohen’s dz = -0.50), or between the AP and ML conditions (mean difference = -0.010, p = 1.000, Cohen’s dz = -0.14) (**Table 7A**). Given the significant condition × muscle interaction, subsequent Bonferroni-adjusted comparisons were used to identify muscle-specific condition differences.

**Table 7:** Bonferroni-adjusted *post hoc* comparisons based on log10-transformed data.

A. Pairwise comparisons for the main effect of condition				
Comparison	Mean difference (I - J)	95% Confidence interval	P-value	Cohen’s dz
AP vs ST	-0.059	-0.123 to 0.005	0.074	-0.55
ML vs ST	-0.050	-0.108 to 0.008	0.110	-0.5
AP vs ML	-0.010	-0.050 to 0.031	1.000	-0.14

B. Condition comparisons for each muscle					
Muscle	Comparison	Mean difference (I - J)	95% Confidence interval	P-value	Cohen’s dz
RA	AP vs ST	-0.126	-0.229 to -0.023	0.014*	-0.72
RA	ML vs ST	-0.136	-0.228 to -0.043	0.003*	-0.86
RA	AP vs ML	0.010	-0.052 to 0.072	1.000	-0.09
IO/TrA	AP vs ST	-0.108	-0.223 to 0.008	0.073	-0.55
GMed	ML vs ST	0.079	-0.008 to 0.167	0.082	-0.53

A, Pairwise comparisons for the main effect of condition; B, condition comparisons for specific muscles with significant effects or secondary descriptive observations. The mean differences and 95% confidence intervals are based on log10-transformed values. Cohen’s dz represents the standardized mean difference for paired comparisons. RA, rectus abdominis; IO/TrA, internal oblique/transversus abdominis; GMed, gluteus medius. AP, anteroposterior instability; ML, mediolateral instability; ST, stable floor condition. Significant differences are indicated by asterisks; *p < 0.05.

In the muscle-specific *post hoc* comparisons, early RA activity was significantly lower in the AP condition (mean difference = -0.126, p = 0.014, Cohen’s dz = -0.72) and ML condition (mean difference = -0.136, p = 0.003, Cohen’s dz = -0.86) than in the ST condition (**Figure 4**). No significant AP–ML difference was observed for the RA (mean difference = 0.010, p = 1.000, Cohen’s dz = 0.09). No other muscle-specific condition differences reached statistical significance. Non-significant comparisons, including the AP versus ST comparison for the IO/TrA (mean difference = -0.108, p = 0.073, Cohen’s dz = -0.55) and the ML versus ST comparison for the GMed (mean difference = 0.079, p = 0.082, Cohen’s dz = 0.53), were treated as secondary descriptive observations (**Table 7B**; **Figure 4**).

## 4 Discussion

This study examined whether AP and ML support-surface instability influenced early neuromuscular responses of the trunk and hip muscles during rapid squatting. The main findings were that EMG onset latency was shorter in the AP condition than in the ST condition, whereas the condition × muscle interaction was not significant. In contrast, early muscle activity during the first 0–75 ms after EMG onset showed a significant condition × muscle interaction, with significant *post hoc* differences mainly limited to lower RA activity in both unstable conditions than in the ST condition. No clear AP–ML differences were observed in either onset latency or early muscle activity. These findings indicate that unstable support-surface conditions may influence both the timing of muscle activation and early muscle output, while providing limited evidence for direction-specific neuromuscular responses between AP and ML rocking conditions.

The shorter EMG onset latency in the AP condition suggests that rapid squatting on an unstable support surface may alter the timing of preparatory or early neuromuscular activity. Previous studies have shown that trunk EMG onset timing is influenced by task demands, movement characteristics, and postural-control requirements during voluntary movements (Granata and England, 2006; Hodges and Richardson, 1996; Moseley et al., 2002; Saunders et al., 2004; Silfies et al., 2009). Therefore, the present findings are consistent with the view that the timing of trunk muscle activity is not fixed, but is modulated according to the mechanical and temporal constraints of the task. In the AP condition, onset latency was shortened overall, and differences between the AP and ST conditions were also observed for the IO/TrA and ES. This may suggest that, during rapid squatting on a support surface unstable in the anteroposterior direction, muscle activity related to sagittal-plane trunk control and trunk stabilization was prepared earlier. However, because the condition × muscle interaction for onset latency was not significant, these findings cannot be concluded to represent clear muscle-specific or direction-dependent responses. In addition, because movement duration, angular velocity, trunk motion, and center-of-mass displacement were not measured, the possibility remains that participants changed their movement strategy under unstable conditions.

For early muscle activity, a significant condition × muscle interaction was observed, indicating that the influence of support-surface condition was not uniform across all muscles. Previous studies on unstable support surfaces have primarily evaluated EMG amplitude-based indices and have reported that unstable conditions can alter trunk and lower-limb muscle activity and force output during squatting or related exercise tasks (Andersen et al., 2014; Anderson and Behm, 2005; Saeterbakken and Fimland, 2013). Unlike previous studies focusing on mean or integrated EMG amplitude, the present study examined the initial 0–75 ms time window after EMG onset. This time window was considered to capture early neuromuscular output immediately after EMG onset, before voluntary or delayed feedback-based corrections based on movement consequences are fully reflected (Aruin and Latash, 1995; Kurtzer, 2014; Massion, 1992; Scott et al., 2015). The main significant *post hoc* finding was that RA activity was lower in both the AP and ML conditions than in the ST condition. This suggests that early muscle output during rapid movement does not necessarily increase uniformly under unstable conditions, but may be redistributed across muscles according to task constraints.

The lower early RA activity observed in both the AP and ML conditions suggests that the initial output of the anterior trunk muscles may have been reduced during rapid squatting on an unstable support surface. The RA is a superficial anterior trunk muscle that contributes to trunk flexion and may also contribute to trunk fixation and spinal stability through coactivation of the abdominal muscles (Brown et al., 2006; Cholewicki and McGill, 1996; Vera-Garcia et al., 2006). Previous studies have shown that trunk muscle coactivation can increase spinal stiffness and stability, but may also increase spinal compressive loading and reduce the flexibility of motor responses to perturbations (Brown et al., 2006; Granata and Marras, 2000; Vera-Garcia et al., 2006). Therefore, the lower early RA activity under unstable conditions may reflect a response that limited excessive anterior trunk fixation during the initiation phase of rapid squatting. However, because trunk stiffness, joint motion, ground reaction forces, and support-surface motion were not directly measured, this interpretation remains inferential. Accordingly, the reduction in RA activity should be interpreted not as direct evidence of an optimized stabilization strategy, but as a characteristic early muscle-output response observed in the present study.

The limited differences between the AP and ML conditions are an important finding of the present study. Previous studies on postural control have demonstrated direction-dependent postural strategies and muscle coordination patterns in response to support-surface translations or external perturbations (Horak and Nashner, 1986; Ting and McKay, 2007; Winter et al., 1996). Based on these findings, we expected that the AP and ML rocker-board conditions would impose different postural-control demands. However, the task used in the present study differed from experimental paradigms involving external perturbations, as participants performed a cue-triggered bilateral squat on a continuously unstable support surface. Although changing the board orientation mechanically permitted rocking in different directions, no clear AP–ML differences were observed in EMG onset latency or early muscle activity. This suggests that the neuromuscular responses observed in the present study may have reflected the general task demand of maintaining posture while performing a rapid movement on an unstable support surface, rather than responses specific to the direction of rocking. However, because trunk motion, center-of-mass displacement, ground reaction forces, and support-surface motion were not measured, it remains unclear to what extent the AP and ML conditions actually produced distinct postural-control demands. Therefore, the present findings should be interpreted cautiously as early neuromuscular responses during rapid squatting on an unstable support surface, rather than as evidence of direction-specific effects of instability.

The significance of this study lies in its focus on the temporal characteristics of neuromuscular activity during rapid squatting on an unstable support surface. Most previous studies on exercise under unstable conditions have evaluated mean or integrated EMG amplitude, whereas the present study examined EMG onset latency and early activity during the first 0–75 ms after EMG onset. The findings suggest that unstable support-surface conditions may influence both the timing of muscle activation and early muscle output at movement initiation. However, because this study examined acute responses in healthy young men, the results should not be interpreted as evidence of training adaptation or improved neuromuscular control. Rather, the present findings provide basic information on how support-surface instability may influence early neuromuscular responses during a controlled rapid squat task.

This study had some limitations. First, the participants were limited to healthy adult men; therefore, the results cannot be directly generalized to women, older adults, or individuals with a history of injury. Second, this study used a rapid squat task with bilateral support, which differs from tasks requiring stronger direction-dependent control, such as single-leg landing or lateral cutting. Third, because surface electromyography was used, the functional separation of the deep muscles and the influence of crosstalk, particularly for the IO/TrA, could not be completely excluded. Fourth, although a condition × muscle interaction was observed for early muscle activity, clear *post hoc* differences were mainly limited to the RA. Therefore, the direction-specific effects that can be interpreted as differences between the AP and ML conditions may have been limited. Fifth, because kinematic and kinetic variables were not measured, it could not be objectively confirmed whether the two board orientations produced distinct AP and ML postural-control demands. Therefore, the observed responses may reflect both the permitted rocking direction and the more general effect of standing on an unstable support surface. Sixth, movement duration and angular velocity were not quantified. Therefore, interindividual variability and possible differences in movement speed across conditions may have influenced EMG onset latency and early muscle activity, limiting the extent to which the observed differences can be attributed solely to support-surface condition. Seventh, the 20-Hz cutoff frequency was selected empirically and was not independently validated. Because EMG onset estimates can be influenced by signal-processing and detection parameters (Tenan et al., 2017), smoothing of the EMG envelope may have affected threshold-crossing times and the need for manual correction. Future studies should use tasks with stronger direction-specific demands, compare different populations, and integrate kinematic and kinetic analyses.

## 5 Conclusions

The results of this study suggest that support-surface instability can influence early neuromuscular responses during rapid squatting, including EMG onset latency and early muscle activity during the first 0–75 ms after EMG onset. EMG onset latency was shorter in the AP condition than in the ST condition, whereas clear AP–ML differences were not observed. Early muscle activity showed muscle-dependent effects, with significant differences mainly limited to lower RA activity in both unstable conditions compared with the ST condition. These findings indicate that unstable support-surface conditions may influence the timing and early output of trunk and hip muscle activity during rapid squatting, but provide limited evidence for direction-specific neuromuscular responses between AP and ML rocker-board conditions. Because kinematic and kinetic variables were not measured, these findings should be interpreted as acute EMG responses to the present task rather than as direct evidence of optimized postural-control strategies.

## Data Availability

The raw data supporting the conclusions of this article will be made available by the authors, without undue reservation.
